# P-1386. Developing a Diagnostic Strategy for the Diagnosis of Bacterial Vaginosis: A Shift from Subjective to Objective Approach

**DOI:** 10.1093/ofid/ofae631.1562

**Published:** 2025-01-29

**Authors:** Pragati Grover, Sunil Sethi, Rajneesh Dadwal, Amit Sehgal, Aastha Saini, Rashmi Bagga, Seema Chopra, Archana Angrup

**Affiliations:** PGIMER, CHANDIGARH, Delhi, Delhi, India; POST GRADUATE INSTITUTE OF MEDICAL EDUCATION AND RESEARCH, CHANDIGARH, CHANDIGARH, Chhattisgarh, India; POST GRADUATE INSTITUTE OF MEDICAL EDUCATION AND RESEARCH, CHANDIGARH, CHANDIGARH, Chhattisgarh, India; All India Institute of Medical Sciences, New Delhi, NEW DELHI, Delhi, India; POST GRADUATE INSTITUTE OF MEDICAL EDUCATION AND RESEARCH, CHANDIGARH, CHANDIGARH, Chhattisgarh, India; PGIMER, Chandigarh, Chandigarh, Chandigarh, India; POST GRADUATE INSTITUTE OF MEDICAL EDUCATION AND RESEARCH, CHANDIGARH, CHANDIGARH, Chhattisgarh, India; POST GRADUATE INSTITUTE OF MEDICAL EDUCATION AND RESEARCH, CHANDIGARH, CHANDIGARH, Chhattisgarh, India

## Abstract

**Background:**

Bacterial vaginosis (BV), a polymicrobial syndrome of unclear etiology is a major public health problem. The current diagnostic approaches lack standardisation and are not easily reproducible. We aim to develop a diagnostic modality to enhance the accurate diagnosis of BV.

**Figure:**
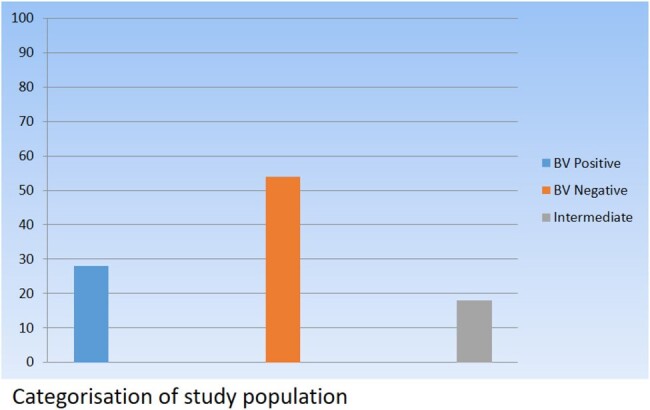

**Methods:**

385 vaginal samples were categorised into BV positive (n=108), BV negative (n=208) and intermediate group (n=69). The positive and negative samples were then subjected to qualitative and quantitative PCR. The two pathogens targetted were *Fannyhessea vaginae* and *Gardenerella vaginalis*.

Standardisation real time PCR: CP value and Tm value for Gardnerella vaginalis

**Figure:**
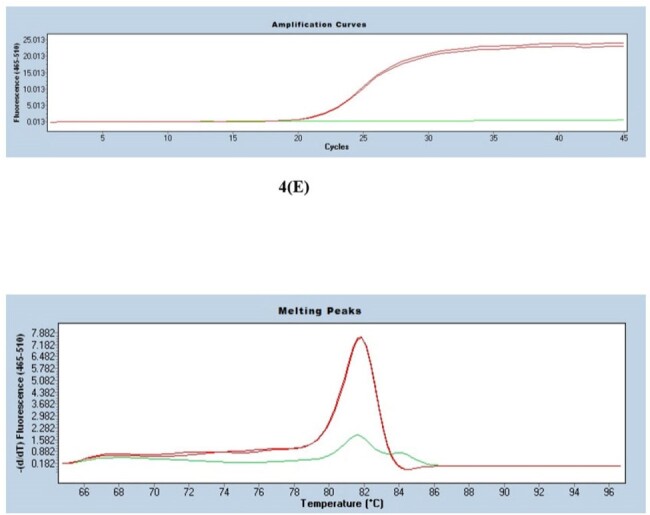

**Results:**

PCR detection as compared to Nugent criteria yielded a sensitivity of 96% and specificity of 82.9%, positive predicitive value (PPV) of 68.5% and negative predicitive value (NPV) of 98.2%. In contrast, molecular quantification of the two bacteria together had highest specificity (99.52%) with excellent PPV of 98.1%. Although, with a low sensitivity (48.1%) and NPV (78%).

**Conclusion:**

All women in reproductive age group should be screened for BV using qualitative PCR targeting *Fannyhessea vaginae* and *Gardenerella vaginalis* together. In symptomatic women, the use of quantitative PCR can provide a consistent diagnosis of BV. Thus this objective diagnostic strategy can play a pivotal role in prevention, early diagnosis, treatment initiation and cure of BV.

**Disclosures:**

**All Authors**: No reported disclosures

